# Supplementary material for: Common adolescent mental health disorders seen in Family Medicine Clinics in Ghana and Nigeria
Source: PLoS One. 2023 Nov 16;18(11):e0285911. doi: 10.1371/journal.pone.0285911 (PMC10653403; doi:10.1371/journal.pone.0285911)
Supplement: S1 Checklist — (DOCX) [file pone.0285911.s001.docx]

STROBE Statement—checklist of items that should be included in reports of observational studies

|  | Item No. | Recommendation | Page  No. | Relevant text from manuscript |
| --- | --- | --- | --- | --- |
| **Title and abstract** | 1 | (*a*) Indicate the study’s design with a commonly used term in the title or the abstract | 1 | A descriptive cross-sectional study involving 302 Physicians practicing in Family Medicine Clinics in Nigeria and Ghana who were randomly selected for the study. |
|  |  | (*b*) Provide in the abstract an informative and balanced summary of what was done and what was found | 1 | **Methodology**  A descriptive cross-sectional study involving 302 Physicians practicing in Family Medicine Clinics in Nigeria and Ghana who were randomly selected for the study. Data were collected using self-administered semi-structured questionnaire, and were entered into excel spreadsheet before analysing with IBM-SPSS version 22. Descriptive statistics using frequencies and percentages was used to describe variables. Ethical approval was obtained prior to commencement of the study.  **Results**  Of the 302 Physicians recruited for the study, only 233 completed the study, in which 168 (72.1%) practiced in Nigeria and 65 (27.9%) in Ghana. They were mostly in urban communities (77.3%) and tertiary health facilities (65.2%). Over 90% of Family Medicine practitioners attended to adolescents with mental health issues with over 70% of them seeing at least 2 adolescents with mental health issues every year. The burden of mental health disorder was 16% and the common mental health disorders seen were depression (59.2%), Bipolar Affective Disorder (55.8%), Epilepsy (51.9%) and Substance Abuse Disorder (44.2%). |
| Introduction | | | |  |
| Background/rationale | 2 | Explain the scientific background and rationale for the investigation being reported | 4 | There is a dearth of mental health experts in West Africa. This is worsened by the stigma associated with mental health disorders in the region. The WHO and World Organization of Family Doctors (WONCA) advocates for the integration of mental health services into primary care as the most viable way of closing the treatment gap and ensuring people get the mental health care they need.^11^ |
| Objectives | 3 | State specific objectives, including any prespecified hypotheses | 4 | The aim of this study was to evaluate the common mental health disorders among adolescents as seen by family Physicians in Family Medicine Clinics in Nigeria and Ghana. |
| Methods | | | |  |
| Study design | 4 | Present key elements of study design early in the paper | 5 | The study was a descriptive cross-sectional study conducted among Family Physicians practising in Nigeria and Ghana from May to September, 2022. |
| Setting | 5 | Describe the setting, locations, and relevant dates, including periods of recruitment, exposure, follow-up, and data collection | 5 | The study sites included General Outpatient Clinics of Teaching Hospitals, Specialist, General and District Hospitals and other Primary Healthcare Centres, where Family Physicians practice in both countries. |
| Participants | 6 | (*a*) *Cohort study*—Give the eligibility criteria, and the sources and methods of selection of participants. Describe methods of follow-up  *Case-control study*—Give the eligibility criteria, and the sources and methods of case ascertainment and control selection. Give the rationale for the choice of cases and controls  *Cross-sectional study*—Give the eligibility criteria, and the sources and methods of selection of participants |  |  |
|  |  | (*b*) *Cohort study*—For matched studies, give matching criteria and number of exposed and unexposed  *Case-control study*—For matched studies, give matching criteria and the number of controls per case |  |  |
| Variables | 7 | Clearly define all outcomes, exposures, predictors, potential confounders, and effect modifiers. Give diagnostic criteria, if applicable | 5 | Other details of the study design and methodology are contained in the published article.^12^ |
| Data sources/ measurement | 8* | For each variable of interest, give sources of data and details of methods of assessment (measurement). Describe comparability of assessment methods if there is more than one group | 5 | Other details of the study design and methodology are contained in the published article.^12^ |
| Bias | 9 | Describe any efforts to address potential sources of bias | 5 | Other details of the study design and methodology are contained in the published article.^12^ |
| Study size | 10 | Explain how the study size was arrived at | 5 | The sample size was calculated to be 302 using fisher’s formula and finite correction was made based on the total population of Family Physicians in each country: 1200 and 125 for Nigeria and Ghana respectively. |

Continued on next page

| Quantitative variables | 11 | Explain how quantitative variables were handled in the analyses. If applicable, describe which groupings were chosen and why | 5 | They were presented in tables and were described using frequencies and percentages. |
| --- | --- | --- | --- | --- |
| Statistical methods | 12 | (*a*) Describe all statistical methods, including those used to control for confounding | 5 | Data were analysed using the Statistical Package for Social Sciences^TM^ (IBM Corp, Armonk, NY, USA) version 22.0. |
|  |  | (*b*) Describe any methods used to examine subgroups and interactions |  |  |
|  |  | (*c*) Explain how missing data were addressed |  | There were no missing data |
|  |  | (*d*) *Cohort study*—If applicable, explain how loss to follow-up was addressed  *Case-control study*—If applicable, explain how matching of cases and controls was addressed  *Cross-sectional study*—If applicable, describe analytical methods taking account of sampling strategy | 5 | Data were analysed using the Statistical Package for Social Sciences^TM^ (IBM Corp, Armonk, NY, USA) version 22.0. |
|  |  | (*e*) Describe any sensitivity analyses |  |  |
| Results | | | | |
| Participants | 13* | (a) Report numbers of individuals at each stage of study—eg numbers potentially eligible, examined for eligibility, confirmed eligible, included in the study, completing follow-up, and analysed | 6 | A total of 233 Family Physicians completed the study (77.2% response rate), in which 65 (27.90%) in Ghana and 168 (72.10%) in Nigeria participated in the study. |
|  |  | (b) Give reasons for non-participation at each stage |  |  |
|  |  | (c) Consider use of a flow diagram |  |  |
| Descriptive data | 14* | (a) Give characteristics of study participants (eg demographic, clinical, social) and information on exposures and potential confounders | 6 | They worked in facilities that were mainly in the urban setting 180 (77.25%). Majority of the facilities were Tertiary institutions 152 (65.24%), which was either Teaching Hospitals or Federal Medical Centres.  The socio-demographic characteristics are shown in Table 1. |
|  |  | (b) Indicate number of participants with missing data for each variable of interest |  | None |
|  |  | (c) *Cohort study*—Summarise follow-up time (eg, average and total amount) |  |  |
| Outcome data | 15* | *Cohort study*—Report numbers of outcome events or summary measures over time |  |  |
|  |  | *Case-control study—*Report numbers in each exposure category, or summary measures of exposure |  |  |
|  |  | *Cross-sectional study—*Report numbers of outcome events or summary measures | *6* | They worked in facilities that were mainly in the urban setting 180 (77.25%). Majority of the facilities were Tertiary institutions 152 (65.24%), which was either Teaching Hospitals or Federal Medical Centres.  The socio-demographic characteristics are shown in Table 1. |
| Main results | 16 | (*a*) Give unadjusted estimates and, if applicable, confounder-adjusted estimates and their precision (eg, 95% confidence interval). Make clear which confounders were adjusted for and why they were included | 7 | The burden of mental health disorder in this study was 16% and the distribution of the common adolescent mental health disorders seen in Family Medicine Clinics in Ghana and Nigeria are as shown in Table 3. Depression 138 (59.23%) was the most commonly seen disorder followed by Bipolar Disorders 130 (55.79%), Epilepsy 121 (51.93%), and Substance Use Disorders 103 (44.21%) in that order. |
|  |  | (*b*) Report category boundaries when continuous variables were categorized |  |  |
|  |  | (*c*) If relevant, consider translating estimates of relative risk into absolute risk for a meaningful time period |  |  |

Continued on next page

| Other analyses | 17 | Report other analyses done—eg analyses of subgroups and interactions, and sensitivity analyses |  |  |
| --- | --- | --- | --- | --- |
| Discussion | | | | |
| Key results | 18 | Summarise key results with reference to study objectives | 8 | Our study showed that 91% of respondents attend to adolescents with mental health issues with over half of them attending to about two to three adolescents with mental health disorders yearly. |
| Limitations | 19 | Discuss limitations of the study, taking into account sources of potential bias or imprecision. Discuss both direction and magnitude of any potential bias | 11 | The study was conducted in two countries in West Africa. Though most Family Medicine Clinics in the region are in these two countries, the results still may not be a true representation of the entire region. Also, the study was conducted among doctors. However, relatively most primary health care centres in the region are run by primary care nurses, community health officers and community health extension workers. These categories of primary care providers were not included in the study even though they attend to most of the patients presenting to primary care facilities in these regions. |
| Interpretation | 20 | Give a cautious overall interpretation of results considering objectives, limitations, multiplicity of analyses, results from similar studies, and other relevant evidence | 8-11 | Substance use disorder and suicide or self-harm were also prevalent among adolescent in this study. This is similar to the findings of Birhanu *et al* in Ethiopia,^24^ Mavura *et al* in northern Tanzania,^25^ and Volkow *et al* in the US.^26^ The reasons may not be unconnected to the high level of peer influence, risk taking behavior and experimentation with substances due to developmental changes and challenges in adolescence.^24,25^ The high burden of self-harm or suicide in this study could be due to the strong relationship between substance abuse and suicide or self-harm especially, among adolescents and young adults.^27,28^ |
| Generalisability | 21 | Discuss the generalisability (external validity) of the study results | 10 | There is an urgent need for Family Physicians to look out for adolescent mental health issues and address them at the early stage before they progress to more complicated forms. There is also the need for policy makers to increase awareness on the burden of mental health disorders among adolescents and put measures in place to mitigate them. |
| Other information | |  | | |
| Funding | 22 | Give the source of funding and the role of the funders for the present study and, if applicable, for the original study on which the present article is based | 11 | Financing of the project was exclusively at the expense of the researchers**.** |

*Give information separately for cases and controls in case-control studies and, if applicable, for exposed and unexposed groups in cohort and cross-sectional studies.

**Note:** An Explanation and Elaboration article discusses each checklist item and gives methodological background and published examples of transparent reporting. The STROBE checklist is best used in conjunction with this article (freely available on the Web sites of PLoS Medicine at http://www.plosmedicine.org/, Annals of Internal Medicine at http://www.annals.org/, and Epidemiology at http://www.epidem.com/). Information on the STROBE Initiative is available at www.strobe-statement.org.
