# Supplementary material for: Common adolescent mental health disorders seen in Family Medicine Clinics in Ghana and Nigeria
Source: PLoS One. 2023 Nov 16;18(11):e0285911. doi: 10.1371/journal.pone.0285911 (PMC10653403; doi:10.1371/journal.pone.0285911)
Supplement: Attachment — Submitted filename: COMMON ADOLESCENT MENTAL HEALTH DISORDERS SEEN IN FAMILY MEDICINE CLINICS IN GHANA AND NIGERIA_reviewed.pdf [file pone.0285911.s003.pdf]

COMMON ADOLESCENT MENTAL HEALTH DISORDERS SEEN IN FAMILY MEDICINE CLINICS IN GHANA AND NIGERIA

General comments: Overall I commend the authors for trying to explore the mental health disorders among adolescents- a vulnerable group of people.

Introduction: Written, however the aims of this study is at variance what was done

Methods: This method can not answer the aims of this study. The study intends to look at mental health disorders among adolescents, yet no adolescent was samples.

Estimated minimum sample size was 302, yet 233 was recruited. Thus, no conclusion can be derived since the analysed sample was not up to the minimum was set that can allowed for conclusion? By the way, what was the power and precision of the study.

A validated questionnaire? No link to this including the previous published work for verification?

“The burden of mental health disorder in this study was 16% and the distribution of the common adolescent mental health disorders seen in Family Medicine Clinics in Ghana and Nigeria are as shown in Table 3”- how was this burden determined, among whom, a snap shot of family physician based on recall? To diagnosis mental disorder of adolescents? Without sampling the adolescents using a standard instrument?
“Depression 138 (59.23%) was the most commonly seen disorder followed by Bipolar Disorders 130 (55.79%), Epilepsy 121 (51.93%), and substance use disorders 103 (44.21%) in that order” -again these are categories of mental disorder with clear guideline for diagnosis/criteria to be fulfilled- check DSM V. Again, this was arrived at based on snap shot respond of family physicians on behalf of adolescents??? This study cannot answer questions on details categories of mental disorders among adolescents, when they themselves were excluded.
The authors urge to have administered standard and validated tools on the adolescents to answers their research questions.
Table 3-how was the diagnosis arrived-based on report of family physicians/hospital records-what of co-existence of more than one conditions.
Conclusion
This research method cannot support the conclusion

Recommendations: Rejected.
