# Supplementary material for: Common adolescent mental health disorders seen in Family Medicine Clinics in Ghana and Nigeria
Source: PLoS One. 2023 Nov 16;18(11):e0285911. doi: 10.1371/journal.pone.0285911 (PMC10653403; doi:10.1371/journal.pone.0285911)
Supplement: Attachment — Submitted filename: PONE COMMON ADOLESCENT MENTAL HEALTH DISORDERS SEEN IN FAMILY_Reviewed.pdf [file pone.0285911.s005.pdf]

PLOS ONE

COMMON ADOLESCENT MENTAL HEALTH DISORDERS SEEN IN FAMILY MEDICINE CLINICS IN GHANA AND NIGERIA

--Manuscript Draft--

**Manuscript Number:**	PONE-D-23-13066R1
**Article Type:**	Research Article
**Full Title:**	COMMON ADOLESCENT MENTAL HEALTH DISORDERS SEEN IN FAMILY MEDICINE CLINICS IN GHANA AND NIGERIA
**Short Title:**	COMMON ADOLESCENT MENTAL HEALTH DISORDERS SEEN IN FAMILY MEDICINE CLINICS IN GHANA AND NIG
**Corresponding Author:**	Tijani Idris Ahmad Oseni, FMCFM<br>Ambrose Alli University<br>Ekpoma, Edo NIGERIA
**Keywords:**	adolescent; mental health; disorder; family physicians; Ghana; Nigeria
**Abstract:**	Background<br>Mental health disorders among adolescents is on the rise globally. For fear of stigmatization, patients seldom present to mental health physicians. They are mostly picked during consultations with Family Physicians. This study seeks to evaluate the common mental health disorders seen by family Physicians in Family Medicine Clinics in Nigeria and Ghana.<br>Methodology<br>A descriptive cross-sectional study involving 302 Physicians practicing in Family Medicine Clinics in Nigeria and Ghana who were randomly selected for the study. Data were collected using self-administered semi-structured questionnaire, and were entered into excel spreadsheet before analysing with IBM-SPSS version 22. Descriptive statistics using frequencies and percentages was used to describe variables. Ethical approval was obtained prior to commencement of the study.<br>Results<br>Of the 302 Physicians recruited for the study, only 233 completed the study, in which 168 (72.1%) practiced in Nigeria and 65 (27.9%) in Ghana. They were mostly in urban communities (77.3%) and tertiary health facilities (65.2%). Over 90% of Family Medicine practitioners attended to adolescents with mental health issues with over 70% of them seeing at least 2 adolescents with mental health issues every year. The burden of mental health disorder was 16% and the common mental health disorders seen were depression (59.2%), Bipolar Affective Disorder (55.8%), Epilepsy (51.9%) and Substance Abuse Disorder (44.2%).<br>Conclusion<br>Family Physicians in Nigeria and Ghana attend to a good number of adolescents with mental health disorders in their Family Medicine clinics. There is the need for Family Physicians to have specialized training and retraining to be able to recognize and treat adolescent mental health disorders. This will help reduce stigmatization and improve the management of the disease thus reducing the burden.
**Order of Authors:**	John Sonny Kumbet
	Tijani Idris Ahmad Oseni, FMCFM
	Magdalene Mensah-Bonsu
	Fatima Mohammed Damagum, MBBS, FWACP
	Edwina Beryl Addo Opare-Lokko
	Eve Namisango
	Abdulgafar Lekan Olawumi
	Onyenwe Chibuike Ephraim
	Benjamin Aweh

Powered by Editorial Manager® and ProduXion Manager® from Aries Systems Corporation

Response to Reviewers:	The manuscript has been revised and a point by point revision attached. Thank you for your comments and hoping for a favourable review of the revised manuscript.
Additional Information:	
Question	Response
**Financial Disclosure**<br><br>Enter a financial disclosure statement that describes the sources of funding for the work included in this submission. Review the submission guidelines for detailed requirements. View published research articles from *PLOS ONE* for specific examples.<br><br>This statement is required for submission and **will appear in the published article** if the submission is accepted. Please make sure it is accurate.<br><br>**Unfunded studies**<br>Enter: *The author(s) received no specific funding for this work.*<br><br>**Funded studies**<br>Enter a statement with the following details:<br>• Initials of the authors who received each award<br>• Grant numbers awarded to each author<br>• The full name of each funder<br>• URL of each funder website<br>• Did the sponsors or funders play any role in the study design, data collection and analysis, decision to publish, or preparation of the manuscript?<br>• **NO** - Include this sentence at the end of your statement: *The funders had no role in study design, data collection and analysis, decision to publish, or preparation of the manuscript.*<br>• **YES** - Specify the role(s) played.<br><br>* typeset	The author(s) received no specific funding for this work.
**Competing Interests**<br><br>Use the instructions below to enter a competing interest statement for this submission. On behalf of all authors, disclose any competing interests that could be perceived to bias this work—acknowledging all financial support and any other relevant financial or non-	The authors have declared that no competing interests exist.

financial competing interests.<br><br>This statement is required for submission and **will appear in the published article** if the submission is accepted. Please make sure it is accurate and that any funding sources listed in your Funding Information later in the submission form are also declared in your Financial Disclosure statement.<br><br>View published research articles from *PLOS ONE* for specific examples.<br><br>**NO authors have competing interests**<br><br>Enter: *The authors have declared that no competing interests exist.*<br><br>**Authors with competing interests**<br><br>Enter competing interest details beginning with this statement:<br><br>*I have read the journal's policy and the authors of this manuscript have the following competing interests: [insert competing interests here]*<br><br>* typeset	
**Ethics Statement**<br><br>Enter an ethics statement for this submission. This statement is required if the study involved:<br><br>• Human participants<br>• Human specimens or tissue<br>• Vertebrate animals or cephalopods<br>• Vertebrate embryos or tissues<br>• Field research<br><br>Write "N/A" if the submission does not require an ethics statement.<br><br>General guidance is provided below. Consult the submission guidelines for	Ethical approval for the study was obtained from the Health Research Ethics Committee of Irrua Specialist Teaching Hospital, Irrua, Nigeria (ISTH/HREC/20221403/275).<br>Written informed consent was obtained from study participants prior to commencement of the study.

detailed instructions. **Make sure that all information entered here is included in the Methods section of the manuscript.**<br><br>**Format for specific study types**<br><br>**Human Subject Research (involving human participants and/or tissue)**<br>• Give the name of the institutional review board or ethics committee that approved the study<br>• Include the approval number and/or a statement indicating approval of this research<br>• Indicate the form of consent obtained (written/oral) or the reason that consent was not obtained (e.g. the data were analyzed anonymously)<br><br>**Animal Research (involving vertebrate animals, embryos or tissues)**<br>• Provide the name of the Institutional Animal Care and Use Committee (IACUC) or other relevant ethics board that reviewed the study protocol, and indicate whether they approved this research or granted a formal waiver of ethical approval<br>• Include an approval number if one was obtained<br>• If the study involved *non-human primates*, add *additional details* about animal welfare and steps taken to ameliorate suffering<br>• If anesthesia, euthanasia, or any kind of animal sacrifice is part of the study, include briefly which substances and/or methods were applied<br><br>**Field Research**<br><br>Include the following details if this study involves the collection of plant, animal, or other materials from a natural setting:<br>• Field permit number<br>• Name of the institution or relevant body that granted permission	
**Data Availability**<br><br>Authors are required to make all data underlying the findings described fully available, without restriction, and from the time of publication. PLOS allows rare	Yes - all data are fully available without restriction

exceptions to address legal and ethical concerns. See the PLOS Data Policy and FAQ for detailed information.<br><br>A Data Availability Statement describing where the data can be found is required at submission. Your answers to this question constitute the Data Availability Statement and **will be published in the article**, if accepted.<br><br>**Important:** Stating 'data available on request from the author' is not sufficient. If your data are only available upon request, select 'No' for the first question and explain your exceptional situation in the text box.<br><br>Do the authors confirm that all data underlying the findings described in their manuscript are fully available without restriction?	
**Describe where the data may be found in full sentences. If you are copying our sample text, replace any instances of XXX with the appropriate details.**<br><br>• If the data are **held or will be held in a public repository**, include URLs, accession numbers or DOIs. If this information will only be available after acceptance, indicate this by ticking the box below. For example: *All XXX files are available from the XXX database (accession number(s) XXX, XXX.*).<br>• If the data are all contained **within the manuscript and/or Supporting Information files**, enter the following: *All relevant data are within the manuscript and its Supporting Information files.*<br>• If neither of these applies but you are able to provide **details of access elsewhere**, with or without limitations, please do so. For example:<br><br>*Data cannot be shared publicly because of [XXX]. Data are available from the XXX Institutional Data Access / Ethics Committee (contact via XXX) for researchers who meet the criteria for*	All relevant data are within the manuscript and its Supporting Information files.

access to confidential data.<br><br>*The data underlying the results presented in the study are available from (include the name of the third party and contact information or URL).*<br>• This text is appropriate if the data are owned by a third party and authors do not have permission to share the data.<br><br>* typeset	
Additional data availability information:	

Powered by Editorial Manager® and ProduXion Manager® from Aries Systems Corporation

Manuscript Click here to access/download;Manuscript;Manuscript.docx

Common Adolescent Mental Health Disorders Seen in Family Medicine Clinics in Ghana and Nigeria

Kumbet John Sonny[1¶], Tijani Idris Ahmad Oseni[2,3¶*], Magdalene Mensah-Bonsu[4¶], Fatima Mohammed Damagum[5¶], Edwina Beryl Addo Opare-Lokko[6,7¶], Eve Namisango[8,9&], AbdulGafar Lekan Olawumi[5&], Onyenwe Chibuike Ephraim[10], Benjamin Aweh[11].

[1] Department of Family Medicine, Geriatric Medicine Sub Division, Federal Medical Centre, Keffi, Nigeria.

[2] Department of Family Medicine, Ambrose Alli University, Ekpoma, Nigeria.

[3] Lifestyle and Behavioural Medicine Unit, Department of Family Medicine, Irrua Specialist Teaching Hospital, Irrua, Nigeria.

[4] Department of Family Medicine, Komfo Anokye Teaching Hospital, Kumasi, Ghana.

[5] Department of Family Medicine, Aminu Kano Teaching Hospital, Kano, Nigeria.

[6] Department of Family Medicine, Greater Accra Regional Hospital, Accra, Ghana.

[7] Faculty of Family Medicine, Ghana College of Physicians and Surgeons, Accra, Ghana.

[8] African Palliative Care Association, Kampala, Uganda.

[9] College of Health Sciences, Makerere University, Kampala, Uganda.

[10] Department of Family Medicine, Alex Ekwueme Federal University Teaching Hospital, Abakaliki, Nigeria.

[11] Department of Mental Health and Behavioural Medicine, Irrua Specialist Teaching Hospital, Irrua, Nigeria.

*** Corresponding Author**: tijanioseni@aauekpoma.edu.ng (TIAO)

¶ These authors contributed equally to this work.

& These authors also contributed equally to this work.

Common Adolescent Mental Health Disorders Seen in Family Medicine Clinics in Ghana and Nigeria

Abstract

Background

Mental health disorders among adolescents is on the rise globally. For fear of stigmatization, patients seldom present to mental health physicians, and they are mostly picked during consultations with Family Physicians. This study seeks to identify the common mental health disorders seen by family Physicians in Family Medicine Clinics in Nigeria and Ghana.

Methods

A descriptive cross-sectional study involving 302 Physicians practicing in Family Medicine Clinics in Nigeria and Ghana, who were randomly selected for the study. Data were collected using self-administered semi-structured questionnaire, and were entered into excel spreadsheet before analysing with IBM-SPSS version 22. Descriptive statistics using frequencies and percentages was used to describe variables. Ethical approval was obtained prior to the commencement of the study.

Results

Of the 302 Physicians recruited for the study, only 233 completed the study, in which 168 (72.1%) practiced in Nigeria and 65 (27.9%) in Ghana. They were mostly in urban communities (77.3%) and tertiary health facilities (65.2%). Over 90% of Family Medicine practitioners attended to

adolescents with mental health issues with over 70% of them seeing at least 2 adolescents with mental health issues every year. The burden of mental health disorder was 16% and the common mental health disorders seen were depression (59.2%), Bipolar Affective Disorder (55.8%), Epilepsy (51.9%) and Substance Abuse Disorder (44.2%).

Conclusion

Family Physicians in Nigeria and Ghana attend to a good number of adolescents with mental health disorders in their clinics. There is the need for Family Physicians to have specialized training and retraining to be able to recognize and treat adolescent mental health disorders. This will help to reduce stigmatization and improve the management of the disease thus, reducing the burden.

Key Words: adolescent, mental health, disorder, burden, family medicine.

Introduction

The burden of mental health disorders is on the rise globally. In 2019, one in every eight people, or 970 million people around the world were living with a mental disorder, in which anxiety and depressive disorders were the most common [1]. In 2020, the amount of people living with anxiety and depressive disorders rose considerably because of the COVID-19 pandemic [1,2].

Adolescents are group of persons with chronological age 10-19 years of age. Adolescence is a critically important stage of life for mental health and well-being of individuals, not only for the reason that this is when young people acquire autonomy, social interaction, self-control, and rapid learning, but also because the abilities and potentials formed in this period have a direct bearing on their mental health for the rest of their lives [3]. Although adolescents generally are highly susceptible to mental health challenges, they receive very little attention, especially in developing countries [4]. Globally, one in seven adolescents experience a mental disorder, accounting for

13% of the global burden of disease in this age group. Depression, anxiety and behavioural disorders are among the leading causes of impairment and disability among them [5].

According to the World Health Organization, common mental disorders such as depression and anxiety account for the largest proportion of mental, developmental and substance use disorders.

Behavioural disorders including attention-deficit and hyperactivity disorder plus conduct disorder are more prevalent among 10-14-year olds, while alcohol and drug use disorders more common in older adolescence (15-19-year olds) [3].

One out of every six young Nigerian aged 15- 24 is suffering from poor mental health, according to a report released by the United Nations Children Fund (UNICEF) [6]. In Ghana, WHO estimated that 650,000 are suffering from severe mental disorder and a further 2,166,000 are suffering from moderate to mild mental disorder with a treatment gap of 98% from the total population [7]. However, the prevalence of mental illness and its burden among adolescents is not known at the national level [8].

There is a dearth of mental health experts in West Africa. This is worsened by the stigma associated with mental health disorders in the region. The World Health Organization (WHO) and World Organization of Family Doctors (WONCA) advocates for the integration of mental health services into primary care as the most viable way of closing the treatment gap and ensuring people get the mental health care they need [9]. The aim of this study is to identify the common mental health disorders among adolescents seen by family Physicians in Family Medicine Clinics in Nigeria and Ghana.

Materials and Methods

Study Design and Setting

The study was a descriptive cross-sectional study conducted among 302 Family Physicians practising in Nigeria and Ghana. It is part of a larger study with some of the findings already published [10]. The sample size was calculated to be 302 using fisher's formula and finite correction was made based on the total population of Family Physicians in each country: 1200 and 125 for Nigeria and Ghana respectively. The sample size (302) was proportionately distributed to each country based on their population size at the time of data collection: Nigeria had 254 while Ghana had 48 Family Physicians. The study sites included General Outpatient Clinics of Teaching Hospitals, Specialist, General and District Hospitals and other Primary Healthcare Centers, where Family Physicians practice in both countries. The consenting Physicians were recruited into the study using multi-stage sampling methods. The questionnaire was in two parts: 1) Sociodemographic variables of the physicians themselves and 2) Data obtained from the family physicians' clinic over six weeks. The physicians reported the number of mental health conditions seen in adolescents over the past six weeks from their clinical records. Other details of the study design and methodology are contained in the published article [10].

Statistical Analysis

Data were analysed using the Statistical Package for Social Sciences^{TM} (IBM Corp, Armonk, NY, USA) version 22.0. They were presented in tables and were described using frequencies and percentages.

Ethical Consideration

Ethical approval for the study was obtained from the Health Research Ethics Committee of Irrua Specialist Teaching Hospital, Irrua, Nigeria (ISTH/HREC/20221403/275).

Results

A total of 233 Family Physicians completed the study (77.2% response rate), in which 65 (27.9%) in Ghana and 168 (72.10%) in Nigeria participated in the study. They worked in facilities that were mainly in the urban setting 180 (77.25%). Majority of the facilities were Tertiary institutions 152 (65.24%), which was either Teaching Hospitals or Federal Medical Centres.

The socio-demographic characteristics are shown in Table 1.

Table 1: Socio-demographic characteristics of respondents (N=233)

VARIABLE	FREQUENCY	PERCENTAGE
**Country**		
Ghana	65	27.90
Nigeria	168	72.10
**Location of Health Facility**		
Rural	53	22.75
Urban	180	77.25
**Type of Facility**		
General/District Hospital	55	23.61
Teaching Hospital/Federal Medical Centre	152	65.24
Private/Mission Hospital	26	11.15

Table 2 shows the adolescent mental health disorders seen in Family Medicine Clinics in Ghana and Nigeria. Over 90% of Family Medicine practitioners attend to adolescents with mental health issues with over 70% of them seeing at least 2 adolescents with mental health issues every year.

Table 2: Adolescent mental health disorders seen in primary care clinics (N=233)

VARIABLE	FREQUENCY	PERCENTAGE
**Do you attend to adolescents with mental health issues? (n=233)**		

Yes	212	90.99
No	21	9.01
**How often do you attend to adolescents with mental health issues? (n=212)**		
Often (> 3 patients a year)	33	15.57
Sometimes (2 – 3 patients a year)	117	55.19
Rarely (≤ 1 patient a year)	62	29.24

The burden of mental health disorder in this study was 16% and the distribution of the common adolescent mental health disorders seen in Family Medicine Clinics in Ghana and Nigeria are as shown in Table 3. Depression 138 (59.23%) was the most commonly seen disorder followed by Bipolar Disorders 130 (55.79%), Epilepsy 121 (51.93%), and Substance Use Disorders 103 (44.21%) in that order.

Table 3: Common adolescent mental disorders seen in primary care clinics (N=233)

**DISORDER**	**FREQUENCY**	**PERCENTAGE**
Depression	138	59.23
Substance Use Disorders	103	44.21
Bipolar Disorders	130	55.79
Psychosis	52	22.32
Suicide or Self Harm	71	30.47
Schizoaffective Disorder	58	24.89
ADHD	39	16.74
Epilepsy	121	51.93
Enuresis	29	12.45

Discussion

The study participants and their distribution have already been described in another publication from the study [10]. Our study showed that 91% of respondents attend to adolescents with mental

health issues with over half of them attending to about two to three adolescents with mental health disorders yearly. This is worrisome, it shows that the burden of adolescent mental health in primary care is enormous. If not attended to, it usually leads to major mental problems including suicide and self-harm which have been on the rise among adolescents [11,12]. It calls for improved capacity in the diagnosis and management of mental health disorders among Family Physicians. This is particularly so as patients hardly present for care as they are generally stigmatized, shunned and denied access to care by their families, caregivers and the society [13]. The burden of mental health disorders among adolescents in primary care in west Africa in this study (16%) is similar to the Global findings of 14% burden of mental health disorders among adolescents [5] and 16% burden found by Robert et al in England [14]. The high burden found in this study can be attributed to the large number ~~of presentation~~ of adolescent to primary care centres as compared to specialist clinics, the fear of stigmatization of mental health disorders and the recent development of subspecialty clinics such as adolescent clinics, and geriatric clinics in General outpatient clinics [3,8].

Respondents reported a variety of mental health disorders seen. The most common disorder was depression. This was followed by bipolar disorder, epilepsy and substance use disorders with the least common disorders as enuresis, Attention Deficit Hyperactivity Disorder (ADHD), Psychosis and Schizoaffective Disorders. World Health Organization (WHO) report that 12 billion work hours and 1 trillion US dollars are lost annually to depression and anxiety alone [15]. On a global scale WHO stated in 2021 that "Depression, anxiety and behavioural disorders are among the leading causes of illness and disability among adolescents" [16], which is in tandem with this study having 59.23% of the respondents treating depression. However, a study done in Nigeria and Ghana [8,17] had Schizophrenia and Epilepsy as the commonest mental health disorders in Nigeria

and Ghana respectively. The above two studies were not done in primary care setting and Providers and stakeholders had limited or no training in adolescent mental health and that could be the reason for the slight difference. The high number of depressions among adolescent can be explained by the high level of poverty and few numbers of specialist to attend to the huge burden of mental health disorder among adolescents [3].

Bipolar disorder was the second leading mental health disorder identified in this study. This could be due to the fact that the most frequent range of onset of bipolar disorder is between the ages of 14-21 years; which falls with the adolescent and early adult age group [18].

In this study, epilepsy was placed third among adolescent mental illnesses. This is not surprising given that, compared to all other continents, Africa has the highest prevalence of epilepsy in children and adolescents [19]. According to a meta-analysis, there are 13–15 new cases of epilepsy for every 1000 people in West Africa [20]. This was attributed to the high prevalence of parasitic and infectious diseases in Africa [19]. The influence of epileptogenic recreational drugs and substances; which are commonly used in this age group could also explain the high burden of seizure disorder in them [19]. The frequent exposure to computer screens, phone screens and flashing lights may also predispose the adolescents to photosensitive epilepsies; which had been reported to be high among them [21].

Substance use disorder and suicide or self-harm were also prevalent among adolescent in this study. This is similar to the findings of Birhanu *et al* in Ethiopia [22], Mavura *et al* in northern Tanzania [23], and Volkow *et al* in the US [24]. The reasons may not be unconnected to the high level of peer influence, risk taking behaviour and experimentation with substances due to developmental changes and challenges in adolescence [22,23]. The high burden of self-harm or

suicide in this study could be due to the strong relationship between substance abuse and suicide or self-harm especially, among adolescents and young adults [25,26].

There is an urgent need for Family Physicians to look out for adolescent mental health issues and address them at the early stage before they progress to more complicated forms. There is also the need for policy makers to increase awareness on the burden of mental health disorders among adolescents and put measures in place to mitigate them.

Conclusion

The prevalence of mental disorders among adolescents seen by Family Physicians in Family Medicine clinics are high in Nigeria and Ghana. There is the need for Family Physicians to have specialized training and retraining on mental health issues concerning adolescents. There is also a need to have more subspecialty adolescent clinics in Family Medicine Clinics to be able to handle adolescents' challenges including recognising and treating adolescent mental health disorders easily. There is also the need for high index of suspicion for these disorders in adolescents when they present. Future studies should seek to establish the relationship between specific variables and types of mental health conditions as well as to establish the background knowledge of health workers in recognition of these conditions. Policy makers should also put measures in place to improve awareness and care for patients.

Limitations

The study was conducted in two countries in West Africa. Though most Family Medicine Clinics in the region are in these two countries, the results still may not be a true representation of the entire region. Also, the study was conducted among doctors. However, relatively most primary health care centres in the region are run by primary care nurses, community health officers and

community health extension workers. These categories of primary care providers were not included in the study even though they attend to most of the patients presenting to primary care facilities in these regions. Being an online survey could explain the high non-response rate of 22.8%.

Author Contribution

KJS, TIAO, MMB, FMD and EBAO conceived the idea, designed the study and conducted the study. EN and ALO analysed the data. All authors wrote the manuscript, edited and revised the manuscript as well as approved the final copy of the manuscript.

Funding

Financing of the project was exclusively at the expense of the researchers**.**

Conflicts of Interests

The researchers declare that there is no conflict of interest whatsoever.

Acknowledgement

We thank Afriwon Research Group ARG of Afriwon Renaissance for bringing young researchers in Africa together and providing the platform from which the research was conducted.

References

1. Institute of Health Metrics and Evaluation. Global Health Data Exchange (GHDx). Global burden of disease 2019. Available from: https://www.vizhub.healthdata.org/gbd-results/. [Accessed 14th May 2022].
2. World Health Organization. Mental Health and COVID-19: Early evidence of the pandemic's impact. Geneva: World Health Organization; 2022. Available from:

https://www.who.int/publications/i/WHO-2019-nCoV-Sci_Brief-Mental_health-2022.1. [Accessed 16th February 2023].

3. World Health Organization. Adolescent mental health in the WHO European Region. 2018. Available from https://www.euro.who.int/en/health-topics/noncommunicable-diseases/mental-health/data-and-resources/fact-sheet-adolescent-mental-health-in-the-who-european-region-2018. [Accessed 3rd March 2022].

4. Addy ND, Agbozo F, Runge-Ranzinger S, Grys P. Mental health difficulties, coping mechanisms and support systems among school-going adolescents in Ghana: A mixed-methods study. *PLoS One* 2021;16(4):e0250424. doi: 10.1371/journal.pone.0250424. eCollection 2021.

5. World Health Organization. Adolescent mental health: Mapping actions of nongovernmental organizations and other international development organizations (2012). Available from https://www.apps.who.int/iris/handle/10665/44875. [Accessed 16th February 2023].

6. UNICEF. The State of the World's Children 2021: On My Mind-Promoting, Protecting and Caring for Children's Mental Health. Available from https://www.unicef.org/reports/state-worlds-children-2021. [Accessed 16th February 2023].

7. Latey A. Leveraging Mobile Phone Technology to Provide Affordable and Accessible Mental Healthcare in Ghana; the MindIT Mental Health Service Story- Harvard Global Health Institute 2020. Available from: https://www.globalhealth.harvard.edu/mindit-mental-health-service-story-ghana/. [Accessed 11th March 2023].

8. Formentos A, Ae-Ngibise KA, Nyame S, Asante KP. Situational analysis of service provision for adolescents with mental and neurological disorders in in two districts of Ghana. *Intl J Mental Health Sys* 2021;15(1):1-21.

9. World Health Organization & World Organization of Family Doctors (2008). Integrating mental health into primary care: a global perspective. World Health Organization. https://apps.who.int/iris/handle/10665/43935. [Accessed 16th February 2023]

10. Oseni TIA, Mensah-Bonsu M, Damagun FM, Salam TO, Sonny KJ, Opare-Lokko EB, et al. Ability and Preparedness of Family Physicians to Recognise and Treat Adolescent Mental Health Disorders in Nigeria and Ghana. *Health Services Insights* 2023;16:11786329231166366.

11. Patton GC, Sawyer SM, Santelli JS, Ross DA, Afifi R, Allen NB, et al. Our future: A Lancet commission on adolescent health and wellbeing. *The Lancet* 2016;387(10036):2423–2478.

12. Beckman K, Mittendorfer-Rutz E, Lichtenstein P, Larsson H, Almqvist C, Runeson B, et al. Mental illness and suicide after self-harm among young adults: long-term follow-up of self-harm patients, admitted to hospital care, in a national cohort. Psychological Medicine. 2016;46(16):3397–405.

13. World Health Organization. Non-communicable diseases 2018. Available from https://www.euro.who.int/en/health-topics/noncommunicable-diseases/mental-health/data-and-resources. [Accessed 10th March 2022]

14. Roberts J, Lenton P, Keetharuth AD, Brazier J. Quality of life impact of mental health conditions in England: results from the adult psychiatric morbidity surveys. *Health and quality of life outcomes* 2014;12(1):1-0.

15. World Health Organization. Mental Health and Substance Use. 2022. Accessed 2022 Mar 10. Available from https://www.who.int/teams/mental-health-and-substance-use/promotion-prevention/mental-health-in-the-workplace. [Accessed 10th March 2022]

16. World Health Organization. Helping adolescents thrive toolkit: strategies to promote and protect adolescent mental health and reduce self-harm and other risk behaviours. Available from:

https://apps.who.int/iris/bitstream/handle/10665/341327/9789240025554-eng.pdf?sequence=1. [Accessed 16th February 2023]

17. Ogbonna PN, Iheanacho PN, Ogbonnaya NP, Mbadugha CJ, Ndubuisi I, Chikeme PC. Prevalence of mental illness among adolescents (15-18 years) treated at Federal Neurospsychiatric Hospital, Enugu Nigeria, from 2004 to 2013. *Arch Psychiatr Nurs* 2020;(1):7-13. doi: 10.1016/j.apnu.2019.12.008. Epub 2019 Dec 13. PMID: 32035592.
18. National Institute of Health. Mental Health Information, 2023 revision: Bipolar disorder in children and teens. Available from https://www.nimh.nih.gov/health/publications/bipolar-disorder-in-children-and-teens. [Accessed 28th April 2023].
19. Olowe-Taiwo R, Shekh-Ahmad T. Pediatric Epilepsy in West Africa: Prevalence, Causes, and Management [Internet]. *Epilepsy - Seizures without Triggers. IntechOpen*; 2023. Available from: http://dx.doi.org/10.5772/intechopen.108255. [Accessed 29th April 2023].
20. Meyer AC, Dua T, Ma J, Saxena S, Birbeck G. Global disparities in the epilepsy treatment gap: A systematic review. *Bulletin of the World Health Organization* 2010;88(4):260-266.
21. Lu Y, Waltz S, Stenzel K, Muhle H, Stephani U. Photosensitivity in epileptic syndromes of childhood and adolescence. *Epileptic Disord* 2008;10(2):136-43. doi: 10.1684/epd.2008.0183. PMID: 18539564.
22. Birhanu AM, Bisetegn TA, Woldeyohannes SM. High prevalence of substance use and associated factors among high school adolescents in Woreta Town, Northwest Ethiopia: multi-domain factor analysis. *BMC Public Health* 2014;14:1186. https//doi.org/10.1186/1471-2458-14-1186.
23. Mavura RA, Nyaki AY, Leyaro BJ, Mamseri R, George J, Ngocho JS, Mboya IB. Prevalence of substance use and associated factors among secondary school adolescents in Kilimanjaro region,

northern Tanzania. *PLoS One* 2022;17(9):e0274102. doi: 10.1371/journal.pone.0274102. PMID: 36054121; PMCID: PMC9439258.

24. Volkow ND, Han B, Einstein EB, Compton WM. Prevalence of Substance Use Disorders by Time Since First Substance Use Among Young People in the US. *JAMA Pediatr.* 2021;175(6):640–643. doi:10.1001/jamapediatrics.2020.6981.

25. Breet E, Bantjes J, Lewis I. Substance use and self-harm: a cross-sectional study of the prevalence, correlates and patterns of medical service utilisation among patients admitted to a South African hospital. *BMC Health Serv Res* 2018;18:157. https://doi.org/10.1186/s12913-018-2963-7.

26. Gupta R, Narnoli S, Das N, Sarkar S, Balhara YPS. Patterns and predictors of self-harm in patients with substance-use disorder. *Indian J Psychiatry* 2019;61(5):431-438. doi: 10.4103/psychiatry.IndianJPsychiatry_578_18.

Click here to access/download
Supporting Information
strobe-statement.org.docx

Data set

Click here to access/download
Supporting Information
Adolescent Mental Health Results.xlsx

Common Adolescent Mental Health Disorders Seen in Family Medicine Clinics in Ghana and Nigeria

Kumbet John Sonny[1¶], Tijani Idris Ahmad Oseni[2,3¶*], Magdalene Mensah-Bonsu[4¶], Fatima Mohammed Damagum[5¶], Edwina Beryl Addo Opare-Lokko[6,7¶], Eve Namisango[8,9&], AbdulGafar Lekan Olawumi[5&], Onyenwe Chibuike Ephraim[10], Benjamin Aweh[11].

[1] Department of Family Medicine, Geriatric Medicine Sub Division, Federal Medical Centre, Keffi, Nigeria.

[2] Department of Family Medicine, Ambrose Alli University, Ekpoma, Nigeria

[3] Lifestyle and Behavioural Medicine Unit, Department of Family Medicine, Irrua Specialist Teaching Hospital, Irrua, Nigeria.

[4] Department of Family Medicine, Komfo Anokye Teaching Hospital, Kumasi, Ghana.

[5] Department of Family Medicine, Aminu Kano Teaching Hospital, Kano, Nigeria.

[6] Department of Family Medicine, Greater Accra Regional Hospital, Accra, Ghana

[7] Faculty of Family Medicine, Ghana College of Physicians and Surgeons, Accra, Ghana.

[8] African Palliative Care Association,

[9] College of Health Sciences, Makerere University, Kampala, Uganda

[10] Department of Family Medicine, Alex Ekwueme Federal University Teaching Hospital, Abakaliki, Nigeria

[11] Department of Mental Health and Behavioural Medicine, Irrua Specialist Teaching Hospital, Irrua, Nigeria.

*** Corresponding Author**: tijanioseni@aauekpoma.edu.ng (TIAO)

Field Code Changed

¶ These authors contributed equally to this work.

Formatted: English (United Kingdom)

& These authors also contributed equally to this work.

Common Adolescent Mental Health Disorders Seen in Family Medicine Clinics in Ghana and Nigeria

Abstract

~~COMMON ADOLESCENT MENTAL HEALTH DISORDERS SEEN IN FAMILY MEDICINE CLINICS IN GHANA AND NIGERIA~~

~~ABSTRACT~~

Background

Formatted: Font: 16 pt

Mental health disorders among adolescents is on the rise globally. For fear of stigmatization, patients seldom present to mental health physicians, and they are mostly picked during consultations with Family Physicians. This study seeks to evaluate the common mental health disorders seen by family Physicians in Family Medicine Clinics in Nigeria and Ghana.

Methods ~~ology~~

Formatted: Font: 16 pt

A descriptive cross-sectional study involving 302 Physicians practicing in Family Medicine Clinics in Nigeria and Ghana, who were randomly selected for the study. Data were collected using self-administered semi-structured questionnaire, and were entered into excel spreadsheet before analysing with IBM-SPSS version 22. Descriptive statistics using frequencies and percentages was used to describe variables. Ethical approval was obtained prior to the commencement of the study.

Results

Formatted: Font: 16 pt

Of the 302 Physicians recruited for the study, only 233 completed the study, in which 168 (72.1%) practiced in Nigeria and 65 (27.9%) in Ghana. They were mostly in urban communities (77.3%) and tertiary health facilities (65.2%). Over 90% of Family Medicine practitioners attended to adolescents with mental health issues with over 70% of them seeing at least 2 adolescents with mental health issues every year. The burden of mental health disorder was 16% and the common mental health disorders seen were depression (59.2%), Bipolar Affective Disorder (55.8%), Epilepsy (51.9%) and Substance Abuse Disorder (44.2%).

Conclusion

Formatted: Font: 16 pt

Family Physicians in Nigeria and Ghana attend to a good number of adolescents with mental health disorders in their ~~Family Medicine~~ clinics. There is the need for Family Physicians to have specialized training and retraining to be able to recognize and treat adolescent mental health disorders. This will help to reduce stigmatization and improve the management of the disease thus, reducing the burden.

Key Words: adolescent, mental health, disorder, burden, family medicine.

Introduction

Formatted: Font: 18 pt

The burden of mental health disorders is on the rise globally. In 2019, ~~1~~ one in every eight ~~8~~ people, or 970 million people around the world were living with a mental disorder, in which ~~with~~ anxiety and depressive disorders were the most common [1].~~[1]~~ In 2020, the amount of people living with anxiety and depressive disorders rose considerably because of the COVID-19 pandemic [1,2].~~[1,2]~~ Adolescents are group of persons with chronological age 10-19 years of age. Adolescence is a critically important stage of life for mental health and well-being of individuals, not only for the reason that this is when young people acquire autonomy, social interaction, self-control, and rapid

learning, but also because the abilities and potentials formed in this period have a direct bearing on their mental health for the rest of their lives [3].[3] Although adolescents generally are highly susceptible to mental health challenges, they receive very little attention, especially in developing countries [4].[4] Globally, one in seven of adolescents experiences a mental disorder, accounting for 13% of the global burden of disease in this age group. with d Depression, anxiety and behavioural disorders are among the leading causes of impairment illness and disability among them [5].[5] According to the World Health Organization, common mental disorders such as depression and anxiety account for the largest proportion of mental, developmental and substance use disorders. Behavioural disorders including attention-deficit and hyperactivity disorder plus conduct disorder are more prevalent among 10-14-year olds, while alcohol and drug use disorders become more common in older adolescence (15-19-year olds) [3]. The total number in both age groups combined is 17 million young people, equivalent to almost 20% or one in five of the population in this age group.[3]

One out of every six young Nigerian aged 15- 24 is suffering from poor mental health, according to a report released by the United Nations Children Fund (UNICEF) [6].[6] In Ghana, WHO estimated that 650,000 are suffering from a severe mental disorder and a further 2,166,000 are suffering from a moderate to mild mental disorder and the with a treatment gap of is 98% from of the total population [7].[7] However, the prevalence of mental illness and its burden among adolescents is not known at on a the national level [8].[8]

There is a dearth of mental health experts in West Africa. This is worsened by the stigma associated with mental health disorders in the region. The World Health Organization (WHO) and World Organization of Family Doctors (WONCA) advocates for the integration of mental health services into primary care as the most viable way of closing the treatment gap and ensuring people get the

Formatted: English (United Kingdom)

mental health care they need [9].[9] The aim of this study is to identify was to evaluate the common mental health disorders among adolescents as seen by family Physicians in Family Medicine Clinics in Nigeria and Ghana.

Materials and Methods

Study Design and Setting

The study was a descriptive cross-sectional study conducted among 302 Family Physicians practising in Nigeria and Ghana. It is part of a larger study with some of the findings already published [10].[10] The sample size was calculated to be 302 using fisher's formula and finite correction was made based on the total population of Family Physicians in each country: 1200 and 125 for Nigeria and Ghana respectively. The sample size (302) was proportionately distributed to each country based on their population size at the time of data collection: Nigeria had 254 while Ghana had 48 Family Physicians. The study sites included General Outpatient Clinics of Teaching Hospitals, Specialist, General and District Hospitals and other Primary Healthcare Centers, where Family Physicians practice in both countries. The consenting Physicians were recruited into the study using multi-stage sampling methods. The questionnaire was in two parts: 1) Sociodemographic variables of the physicians themselves and 2) Data obtained from the family physicians' clinic over six weeks. The physicians reported the number of mental health conditions seen in adolescents over the past six weeks from their clinical records. Other details of the study design and methodology are contained in the published article [10].A total of 302 Physicians Practicing in Family Medicine Clinics in Nigeria and Ghana who consented to the study were randomly selected for the study using a multistage sampling technique. Two countries in West Africa (Nigeria and Ghana) were selected using purposive sampling. Simple random sampling was then used to select Family Medicine clinics across both countries and all the Family Physicians in

Formatted: Font: 18 pt

Formatted: Font: 18 pt

Formatted: Font: Bold

Formatted: Font: 16 pt

Formatted: English (United Kingdom)

the selected clinics that met the selection criteria were recruited for the study. Those included were Consultants/Senior Specialists, Specialists/Senior Registrars/Registrars and Medical Officers in Family Medicine Clinics in both countries. Those who were not currently practising were excluded from the study. The sample size was determined using fisher's formula, with 50.0% used in the absence of previously reported proportion. The Association of General and Private Medical Practitioners in Nigeria (AGPMPN) and the Society of Family Physicians of Ghana estimates that there are currently 1200 and 125 Family Physicians in Nigeria and Ghana respectively. Thus, the sample size for finite population was determined to be 302. Proportionate sampling was then used to determine the sample size for Nigeria and Ghana to be 254 and 48 respectively. However, 233 physicians: 168 and 65 from Nigeria and Ghana respectively responded to the study. This gave a response rate of 77.2% with a country response rate of 66.1% for Nigeria and 135% for Ghana. Physicians from Ghana cooperated more hence more were recruited to increase the power and compensate to some extent for the poor response from Nigeria.

A semi-structured self-administered questionnaire containing information on sociodemographic variables, information on mental health disorders seen among adolescents as well as information on the ability and preparedness of respondents to recognise and treat mental health disorders among adolescents was used to obtain information from respondents electronically. The questionnaire was developed by the researchers in line with the objectives and evaluated by a team of experts in Family Medicine and Mental Health for face and content validity and reliability. A Cronbach's alpha coefficient of 0.85 was obtained. This is indicative of high dependability of the instrument. It was pretested among medical practitioners of the staff clinic of Irrua Specialist Teaching Hospital, Irrua before commencement of the study. The database of the Association of General and Private Medical Practitioners in Nigeria (AGPMPN), Society of Family Physicians

~~of Nigeria (SOFPON) and that of Ghana as well as training centres accredited for the training of Family Physicians in Nigeria and Ghana was used in identifying and selecting respondents. Questionnaires were sent to study participants through Email and WhatsApp. Responses were received through same channels.~~

Statistical Analysis

Formatted: Font: 16 pt

Data were analysed using the Statistical Package for Social Sciences™ (IBM Corp, Armonk, NY, USA) version 22.0. They were presented in tables and were described using frequencies and percentages.

Ethical Consideration

Formatted: Font: 16 pt

Ethical approval for the study was obtained from the Health Research Ethics Committee of Irrua Specialist Teaching Hospital, Irrua, Nigeria (ISTH/HREC/20221403/275).

Formatted: English (United Kingdom)

Results

Formatted: Font: 18 pt

A total of 233 Family Physicians completed the study (77.2% response rate), in which 65 (27.9%) in Ghana and 168 (72.10%) in Nigeria participated in the study. They worked in facilities that were mainly in the urban setting 180 (77.25%). Majority of the facilities were Tertiary institutions 152 (65.24%), which was either Teaching Hospitals or Federal Medical Centres.

The socio-demographic characteristics are shown in Table 1.

Table 1: Socio-demographic characteristics of respondents (N=233)

VARIABLE	FREQUENCY	PERCENTAGE
**Country**		

Ghana	65	27.90
Nigeria	168	72.10
**Location of Health Facility**		
Rural	53	22.75
Urban	180	77.25
**Type of Facility**		
General/District Hospital	55	23.61
Teaching Hospital/Federal Medical Centre	152	65.24
Private/Mission Hospital	26	11.15

Table 2 shows the adolescent mental health disorders seen in Family Medicine Clinics in Ghana and Nigeria. Over 90% of Family Medicine practitioners attend to adolescents with mental health issues with over 70% of them seeing at least 2 adolescents with mental health issues every year.

Table 2: Adolescent mental health disorders seen in primary care clinics (N=233)

VARIABLE	FREQUENCY	PERCENTAGE
**Do you attend to adolescents with mental health issues? (n=233)**		
Yes	212	90.99
No	21	9.01
**How often do you attend to adolescents with mental health issues? (n=212)**		
Often (> 3 patients a year)	33	15.57
Sometimes (2 – 3 patients a year)	117	55.19
Rarely (≤ 1 patient a year)	62	29.24

The burden of mental health disorder in this study was 16% and the distribution of the common adolescent mental health disorders seen in Family Medicine Clinics in Ghana and Nigeria are as shown in Table 3. Depression 138 (59.23%) was the most commonly seen disorder followed by

Bipolar Disorders 130 (55.79%), Epilepsy 121 (51.93%), and Substance Use Disorders 103 (44.21%) in that order.

Table 3: Common adolescent mental disorders seen in primary care clinics (N=233)

DISORDER	FREQUENCY	PERCENTAGE
Depression	138	59.23
Substance Use Disorders	103	44.21
Bipolar Disorders	130	55.79
Psychosis	52	22.32
Suicide or Self Harm	71	30.47
Schizoaffective Disorder	58	24.89
ADHD	39	16.74
Epilepsy	121	51.93
Enuresis	29	12.45

Discussion

Formatted: Font: 18 pt

The study participants and their distribution have already been described in another publication from the study [10].~~[10]~~ Our study showed that 91% of respondents attend to adolescents with mental health issues with over half of them attending to about two to three adolescents with mental health disorders yearly. This is worrisome, it shows that the burden of adolescent mental health in primary care is enormous. If not attended to, it usually leads to major mental problems including suicide and self-harm which have been on the rise among adolescents [11,12].~~[11,12]~~ It calls for improved capacity in the diagnosis and management of mental health disorders among Family Physicians. This is particularly so as patients hardly present for care as they are generally stigmatized, shunned and denied access to care by their families, caregivers and the society [13].~~[13]~~ The burden of mental health disorders among adolescents in primary care in west Africa in this study (16%) is similar to the Global findings of 14% burden of mental health disorders among adolescents [5]~~[5]~~ and 16%

burden found by Robert et al in England [14].[14] The high burden found in this study can be attributed to the large number of presentation of adolescent to primary care centres as compared to specialist clinics, the fear of stigmatization of mental health disorders and the recent development of subspecialty clinics such as adolescent clinics, and geriatric clinics in General outpatient clinics [3,8].[3,8]

Respondents reported a variety of mental health disorders seen. The most common disorder was depression. This was followed by bipolar disorder, epilepsy and substance use disorders with the least common disorders as enuresis, Attention Deficit Hyperactivity Disorder (ADHD), Psychosis and Schizoaffective Disorders. World Health Organization (WHO) report that 12 billion work hours and 1 trillion US dollars are lost annually to depression and anxiety alone [15].[15] On a global scale WHO stated in 2021 that “Depression, anxiety and behavioural disorders are among the leading causes of illness and disability among adolescents” [16],[16] which is in tandem with this study having 59.23% of the respondents treating depression. However, a study done in Nigeria and Ghana [8,17][8,17] had Schizophrenia and Epilepsy as the commonest mental health disorders in Nigeria and Ghana respectively. The above two studies were not done in primary care setting and Providers and stakeholders had limited or no training in adolescent mental health and that could be the reason for the slight difference. The high number of depressions among adolescent can be explained by the high level of poverty and few numbers of specialist to attend to the huge burden of mental health disorder among adolescents [3].[3]

Bipolar disorder was the second leading mental health disorder identified in this study. This could be due to the fact that the most frequent range of onset of bipolar disorder is between the ages of 14-21 years; which falls with the adolescent and early adult age group [18].[18]

In this study, epilepsy was placed third among adolescent mental illnesses. This is not surprising given that, compared to all other continents, Africa has the highest prevalence of epilepsy in children and adolescents [19].~~19~~ According to a meta-analysis, there are 13–15 new cases of epilepsy for every 1000 people in West Africa [20].~~20~~ This was attributed to the high prevalence of parasitic and infectious diseases in Africa [19].~~19~~ The influence of epileptogenic recreational drugs and substances; which are commonly used in this age group could also explain the high burden of seizure disorder in them [19].~~19~~ The frequent exposure to computer screens, phone screens and flashing lights may also predispose the adolescents to photosensitive epilepsies; which had been reported to be high among them [21].~~21~~

Formatted: English (United Kingdom)

Substance use disorder and suicide or self-harm were also prevalent among adolescent in this study. This is similar to the findings of Birhanu *et al* in Ethiopia [22],~~22~~ Mavura *et al* in northern Tanzania [23],~~23~~ and Volkow *et al* in the US [24].~~24~~ The reasons may not be unconnected to the high level of peer influence, risk taking ~~behavior~~behaviour and experimentation with substances due to developmental changes and challenges in adolescence [22,23].~~22,23~~ The high burden of self-harm or suicide in this study could be due to the strong relationship between substance abuse and suicide or self-harm especially, among adolescents and young adults [25,26].~~25,26~~

Formatted: English (United Kingdom)

Formatted: English (United Kingdom)

There is an urgent need for Family Physicians to look out for adolescent mental health issues and address them at the early stage before they progress to more complicated forms. There is also the need for policy makers to increase awareness on the burden of mental health disorders among adolescents and put measures in place to mitigate them.

Conclusion

Formatted: Font: 18 pt

The prevalence of mental disorders among adolescents seen by Family Physicians in Family Medicine clinics are high in Nigeria and Ghana. There is the need for Family Physicians to have

specialized training and retraining on mental health issues concerning adolescents. There is also a need to have more subspecialty adolescent clinics in Family Medicine Clinics to be able to handle adolescents' challenges including recognising and treating adolescent mental health disorders easily. There is also the need for high index of suspicion for these disorders in adolescents when they present. Policy makers should also put measures in place to improve awareness and care for patients.

Limitations

Formatted: Font: 18 pt

The study was conducted in two countries in West Africa. Though most Family Medicine Clinics in the region are in these two countries, the results still may not be a true representation of the entire region. Also, the study was conducted among doctors. However, relatively most primary health care centres in the region are run by primary care nurses, community health officers and community health extension workers. These categories of primary care providers were not included in the study even though they attend to most of the patients presenting to primary care facilities in these regions. Being an online survey could explain the high non-response rate of 22.8%.

Author Contribution

Formatted: Font: 18 pt

KJS~~K~~, ~~O~~TIAO, M~~B~~MB, ~~D~~FMD~~, OLE~~ and EBAO conceived the idea, designed the study and conducted the study. EN~~E~~ and ALO analysed the data. All authors wrote the manuscript, edited and revised the manuscript as well as approved the final copy of the manuscript.

Funding

Formatted: Font: 18 pt

Financing of the project was exclusively at the expense of the researchers.

Conflicts of Interests

The researchers declare that there is no conflict of interest whatsoever.

Acknowledgement

We thank Afriwon Research Group ARG of Afriwon Renaissance for bringing young researchers in Africa together and providing the platform from which the research was conducted.

References

1. Institute of Health Metrics and Evaluation. Global Health Data Exchange (GHDx). Global burden of disease 2019. Available from: https://www.vizhub.healthdata.org/gbd-results/. [Accessed 14th May 2022].
2. World Health Organization. Mental Health and COVID-19: Early evidence of the pandemic's impact. Geneva: World Health Organization; 2022. Available from: https://www.who.int/publications/i/WHO-2019-nCoV-Sci_Brief-Mental_health-2022.1. [Accessed 16th February 2023].
3. World Health Organization. Adolescent mental health in the WHO European Region. 2018. Available from https://www.euro.who.int/en/health-topics/noncommunicable-diseases/mental-health/data-and-resources/fact-sheet-adolescent-mental-health-in-the-who-european-region-2018. [Accessed 3rd March 2022].
4. Addy ND, Agbozo F, Runge-Ranzinger S, Grys P. Mental health difficulties, coping mechanisms and support systems among school-going adolescents in Ghana: A mixed-methods study. *PLoS One* 2021;16(4):e0250424. doi: 10.1371/journal.pone.0250424. eCollection 2021.

Formatted: Font: 18 pt

Formatted: Font: 18 pt

Formatted: Font: 18 pt

Field Code Changed

Field Code Changed

Formatted: No underline, Font color: Auto

Field Code Changed

5. World Health Organization. Adolescent mental health: Mapping actions of nongovernmental organizations and other international development organizations (2012). Available from https://www.apps.who.int/iris/handle/10665/44875. [Accessed 16th February 2023].

6. UNICEF. The State of the World's Children 2021: On My Mind-Promoting, Protecting and Caring for Children's Mental Health. Available from https://www.unicef.org/reports/state-worlds-children-2021. [Accessed 16th February 2023].

7. Latey A. Leveraging Mobile Phone Technology to Provide Affordable and Accessible Mental Healthcare in Ghana; the MindIT Mental Health Service Story- Harvard Global Health Institute 2020. Available from: https://www.globalhealth.harvard.edu/mindit-mental-health-service-story-ghana/. [Accessed 11th March 2023].

8. Formentos A, Ae-Ngibise KA, Nyame S, Asante KP. Situational analysis of service provision for adolescents with mental and neurological disorders in in two districts of Ghana. *Intl J Mental Health Sys* 2021;15(1):1-21.

9. World Health Organization & World Organization of Family Doctors (2008). Integrating mental health into primary care: a global perspective. World Health Organization. https://apps.who.int/iris/handle/10665/43935. [Accessed 16th February 2023]

10. Oseni TIA, Mensah-Bonsu M, Damagun FM, Salam TO, Sonny KJ, Opare-Lokko EB, et al. Ability and Preparedness of Family Physicians to Recognise and Treat Adolescent Mental Health Disorders in Nigeria and Ghana. *Health Services Insights* 2023;16:11786329231166366.

11. Patton GC, Sawyer SM, Santelli JS, Ross DA, Afifi R, Allen NB, et al. Our future: A Lancet commission on adolescent health and wellbeing. *The Lancet* 2016;387(10036):2423–2478.

12. Beckman K, Mittendorfer-Rutz E, Lichtenstein P, Larsson H, Almqvist C, Runeson B, et al. Mental illness and suicide after self-harm among young adults: long-term follow-up of self-harm

patients, admitted to hospital care, in a national cohort. Psychological Medicine. 2016;46(16):3397–405.

13. World Health Organization. Non-communicable diseases 2018. Available from https://www.euro.who.int/en/health-topics/noncommunicable-diseases/mental-health/data-and-resources. [Accessed 10th March 2022]

14. Roberts J, Lenton P, Keetharuth AD, Brazier J. Quality of life impact of mental health conditions in England: results from the adult psychiatric morbidity surveys. *Health and quality of life outcomes* 2014;12(1):1-0.

15. World Health Organization. Mental Health and Substance Use. 2022. Accessed 2022 Mar 10. Available from https://www.who.int/teams/mental-health-and-substance-use/promotion-prevention/mental-health-in-the-workplace. [Accessed 10th March 2022]

16. World Health Organization. Helping adolescents thrive toolkit: strategies to promote and protect adolescent mental health and reduce self-harm and other risk behaviours. Available from: https://apps.who.int/iris/bitstream/handle/10665/341327/9789240025554-eng.pdf?sequence=1. [Accessed 16th February 2023]

17. Ogbonna PN, Iheanacho PN, Ogbonnaya NP, Mbadugha CJ, Ndubuisi I, Chikeme PC. Prevalence of mental illness among adolescents (15-18 years) treated at Federal Neurospsychiatric Hospital, Enugu Nigeria, from 2004 to 2013. *Arch Psychiatr Nurs* 2020;(1):7-13. doi: 10.1016/j.apnu.2019.12.008. Epub 2019 Dec 13. PMID: 32035592.

18. National Institute of Health. Mental Health Information, 2023 revision: Bipolar disorder in children and teens. Available from https://www.nimh.nih.gov/health/publications/bipolar-disorder-in-children-and-teens. [Accessed 28th April 2023].

Field Code Changed

Formatted: No underline, Font color: Auto

Formatted: No underline, Font color: Auto

Field Code Changed

Field Code Changed

Field Code Changed

19. Olowe-Taiwo R, Shekh-Ahmad T. Pediatric Epilepsy in West Africa: Prevalence, Causes, and Management [Internet]. *Epilepsy - Seizures without Triggers*. *IntechOpen*; 2023. Available from: http://dx.doi.org/10.5772/intechopen.108255. [Accessed 29th April 2023].

20. Meyer AC, Dua T, Ma J, Saxena S, Birbeck G. Global disparities in the epilepsy treatment gap: A systematic review. *Bulletin of the World Health Organization* 2010;88(4):260-266.

21. Lu Y, Waltz S, Stenzel K, Muhle H, Stephani U. Photosensitivity in epileptic syndromes of childhood and adolescence. *Epileptic Disord* 2008;10(2):136-43. doi: 10.1684/epd.2008.0183. PMID: 18539564.

22. Birhanu AM, Bisetegn TA, Woldeyohannes SM. High prevalence of substance use and associated factors among high school adolescents in Woreta Town, Northwest Ethiopia: multi-domain factor analysis. *BMC Public Health* 2014;14:1186. https//doi.org/10.1186/1471-2458-14-1186.

23. Mavura RA, Nyaki AY, Leyaro BJ, Mamseri R, George J, Ngocho JS, Mboya IB. Prevalence of substance use and associated factors among secondary school adolescents in Kilimanjaro region, northern Tanzania. *PLoS One* 2022;17(9):e0274102. doi: 10.1371/journal.pone.0274102. PMID: 36054121; PMCID: PMC9439258.

24. Volkow ND, Han B, Einstein EB, Compton WM. Prevalence of Substance Use Disorders by Time Since First Substance Use Among Young People in the US. *JAMA Pediatr*. 2021;175(6):640–643. doi:10.1001/jamapediatrics.2020.6981.

25. Breet E, Bantjes J, Lewis I. Substance use and self-harm: a cross-sectional study of the prevalence, correlates and patterns of medical service utilisation among patients admitted to a South African hospital. *BMC Health Serv Res* 2018;18:157. https://doi.org/10.1186/s12913-018-2963-7.

Formatted: English (United Kingdom)

Field Code Changed

Formatted: English (United Kingdom)

Formatted: English (United Kingdom)

Formatted: English (United Kingdom)

Formatted: English (United Kingdom)

Formatted: English (United Kingdom)

Formatted: English (United Kingdom)

Field Code Changed

Formatted: English (United Kingdom)

26. Gupta R, Narnoli S, Das N, Sarkar S, Balhara YPS. Patterns and predictors of self-harm in patients with substance-use disorder. *Indian J Psychiatry* 2019;61(5):431-438. doi: 10.4103/psychiatry.IndianJPsychiatry_578_18.

Formatted: English (United Kingdom)

Formatted: English (United Kingdom)

Response to reviewers

Editor/Reviewer Number	Original comments of the reviewer	Reply by the author(s)	Changes done on page number and line number
Journal Editor	Please ensure that your manuscript meets PLOS ONE's style requirements, including those for file naming.	Done	Affected pages
	We noticed you have some minor occurrence of overlapping text with the following previous publication(s), which needs to be addressed.<br>In your revision ensure you cite all your sources (including your own works), and quote or rephrase any duplicated text outside the methods section.	Done	Affected pages
	In your Data Availability statement, you have not specified where the minimal data set underlying the results described in your manuscript can be found. PLOS defines a study's minimal data set as the underlying data used to reach the	Data set provided as supporting information file	

	conclusions drawn in the manuscript and any additional data required to replicate the reported study findings in their entirety. All PLOS journals require that the minimal data set be made fully available. Upon re-submitting your revised manuscript, please upload your study's minimal underlying data set as either Supporting Information files or to a stable, public repository and include the relevant URLs, DOIs, or accession numbers within your revised cover letter.		
Reviewer 1	Introduction: The aims of this study is at variance with what was done	Aim modified to align with what was done	Page 5, line 89
	Methods: This method can not answer the aims of this study.<br>The study intends to look at mental health disorders among adolescents, yet no adolescent was samples.<br>Estimated minimum sample size was 302, yet 233 was recruited.	The aim has been modified to align with the methodology.<br><br>The study targeted family physicians who attended to the adolescents with mental health disorders, and not the patients.<br><br>With an overall response rate of 77% and over a 100% response rate from Ghana, the result can be relied upon for the	

	Thus, no conclusion can be derived since the analysed sample was not up to the minimum was set that can allowed for conclusion?<br>By the way, what was the power and precision of the study.<br>A validated questionnaire? No link to this including the previous published work for verification?	conclusion reached. However, it has also been included in the limitations.<br><br>A power of 80% was used.<br><br>The questionnaire was self validated and the process was explained in the previous publication (https://doi.org/10.1177/11786329231166)	
	"The burden of mental health disorder in this study was 16% and the distribution of the common adolescent mental health disorders seen in Family Medicine Clinics in Ghana and Nigeria are as shown in Table 3"- how was this burden determined, among whom, a snap shot of family physician based on recall? To diagnosis mental disorder of adolescents? Without sampling the adolescents using a standard instrument? "Depression 138	The burden was determined based on physicians' recall of the distribution of patients seen and corroborated by clinic records.<br>Diagnosis was made by family physicians using standard clinical diagnostic procedures for mental health disorders. These physicians were professionals who had received basic medical trainings in mental health with some having additional specialist training in mental health as reported in the previous published manuscript (https://doi.org/10.1177/11786329231166).<br>So they had the requisite expertise to have made the various diagnosis and their reports corroborated by patients medical records were sufficient evidence for various categories of mental disorders diagnosed.	Page 5, line 104

	(59.23%) was the most commonly seen disorder followed by Bipolar Disorders 130 (55.79%), Epilepsy 121 (51.93%), and substance use disorders 103 (44.21%) in that order” -again these are categories of mental disorder with clear guideline for diagnosis/criteria to be fulfilled- check DSM V. Again, this was arrived at based on snap shot respond of family physicians on behalf of adolescents??? This study cannot answer questions on details categories of mental disorders among adolescents, when they themselves were excluded.<br>The authors urge to have administered standard and validated tools on the adolescents to answers their research questions.<br>Table 3-how was the diagnosis arrived-based on report of family physicians/hospital		

	records-what of co-existence of more than one conditions.		
	Conclusion<br>This research method cannot support the conclusion	Sir, you may reconsider based on the revisions made and the above explanations.<br>Thank you.	
Reviewer 2	The manuscript will benefit greatly from a thorough language proofreading.	Done	Affected pages
	The objective stated that the study seeks to "evaluate" the common mental health disorders seen...This used of the quoted word should be revised as the mental health disorders were mainly identified	The aim of this study is to "identify" the common mental health disorders.	Page 5, line 89
	Some abbreviations were used in the manuscript which were not specifically defined e.g WHO, PTSD	Corrected	Page 4, line 86<br>Page 10, line 188
	There is a need to ensure that adequate information is provided in "Materials and Methods" section e.g information about the contents (sections) in the data collection tool, how the tools was deployed, validated and the parameters used for the sample	Effected. However, providing most of the requested details would mean repeating what has already being published as details of the methodology were published in a previous paper which was referenced (https://doi.org/10.1177/11786329231166).	Pages 5 line 96

	size calculation. The multi-stage sampling method should be clearly explained		
	Was ethical approval obtained in Ghana? If no, why?	No. The study was an online survey. Ethical approval was sought and obtained from the ethics committee of the institution of the principal investigator and information obtained online from physicians in Nigeria and Ghana.	
	A non-response rate of over 20% was recorded. What was the reason for this? Can it be included in the limitation?	Included in the limitation	Page 12, line 238
	In the results section, a chart showing the common adolescent mental health disorder (by country or location of health facility) should be considered (e.g a clustered column bar chart)	Thank you. However, the authors did not consider it necessary as the study was not a comparative study of Nigeria and Ghana rather it was meant to bring to fore the magnitude of adolescent mental health disorders seen by family physicians in both countries and the need for system strengthening to address them.	
	What further research suggestion on this subject do the authors think should be prioritized? This could be included in the conclusion section.	Done	Page 12, line 229
