# Supplementary material for: Common adolescent mental health disorders seen in Family Medicine Clinics in Ghana and Nigeria
Source: PLoS One. 2023 Nov 16;18(11):e0285911. doi: 10.1371/journal.pone.0285911 (PMC10653403; doi:10.1371/journal.pone.0285911)
Supplement: Attachment — Submitted filename: Response to reviewers.docx [file pone.0285911.s004.docx]

**Response to reviewers**

| Editor/Reviewer Number | Original comments of the reviewer | Reply by the author(s) | Changes done on page number and line number |
| --- | --- | --- | --- |
| Journal Editor | Please ensure that your manuscript meets PLOS ONE's style requirements, including those for file naming. | Done | Affected pages |
|  | We noticed you have some minor occurrence of overlapping text with the following previous publication(s), which needs to be addressed.  In your revision ensure you cite all your sources (including your own works), and quote or rephrase any duplicated text outside the methods section. | Done | Affected pages |
|  | In your Data Availability statement, you have not specified where the minimal data set underlying the results described in your manuscript can be found. PLOS defines a study's minimal data set as the underlying data used to reach the conclusions drawn in the manuscript and any additional data required to replicate the reported study findings in their entirety. All PLOS journals require that the minimal data set be made fully available.  Upon re-submitting your revised manuscript, please upload your study’s minimal underlying data set as either Supporting Information files or to a stable, public repository and include the relevant URLs, DOIs, or accession numbers within your revised cover letter. | Data set provided as supporting information file |  |
| Reviewer 1 | Introduction: The aims of this study is at variance with what was done | Aim modified to align with what was done | Page 5, line 89 |
|  | Methods: This method can not answer the aims of this study.  The study intends to look at mental health disorders among adolescents, yet no adolescent was samples.  Estimated minimum sample size was 302, yet 233 was recruited. Thus, no conclusion can be derived since the analysed sample was not up to the minimum was set that can allowed for conclusion?  By the way, what was the power and precision of the study. A validated questionnaire? No link to this including the previous published work for verification? | The aim has been modified to align with the methodology.  The study targeted family physicians who attended to the adolescents with mental health disorders, and not the patients.  With an overall response rate of 77% and over a 100% response rate from Ghana, the result can be relied upon for the conclusion reached. However, it has also been included in the limitations.  A power of 80% was used.  The questionnaire was self validated and the process was explained in the previous publication (https://doi.org/10.1177/11786329231166) |  |
|  | “The burden of mental health disorder in this study was 16% and the distribution of the common adolescent mental health disorders seen in Family Medicine Clinics in Ghana and Nigeria are as shown in Table 3”- how was this burden determined, among whom, a snap shot of family physician based on recall? To diagnosis mental disorder of adolescents?  Without sampling the adolescents using a standard instrument? “Depression 138 (59.23%) was the most commonly seen disorder followed by Bipolar Disorders 130 (55.79%), Epilepsy 121 (51.93%), and substance use disorders 103 (44.21%) in that order” -again these are categories of mental disorder with clear guideline for diagnosis/criteria to be fulfilled- check DSM V. Again, this was arrived at based on snap shot respond of family physicians on behalf of adolescents??? This study cannot answer questions on details categories of mental disorders among adolescents, when they themselves were excluded. The authors urge to have administered standard and validated tools on the adolescents to answers their research questions. Table 3-how was the diagnosis arrived-based on report of family physicians/hospital records-what of co-existence of more than one conditions. | The burden was determined based on physicians’ recall of the distribution of patients seen and corroborated by clinic records.  Diagnosis was made by family physicians using standard clinical diagnostic procedures for mental health disorders. These physicians were professionals who had received basic medical trainings in mental health with some having additional specialist training in mental health as reported in the previous published manuscript (<https://doi.org/10.1177/11786329231166>).  So they had the requisite expertise to have made the various diagnosis and their reports corroborated by patients medical records were sufficient evidence for various categories of mental disorders diagnosed. | Page 5, line 104 |
|  | Conclusion This research method cannot support the conclusion | Sir, you may reconsider based on the revisions made and the above explanations.  Thank you. |  |
| Reviewer 2 | The manuscript will benefit greatly from a thorough language proofreading. | Done | Affected pages |
|  | The objective stated that the study seeks to "evaluate" the common mental health disorders seen...This used of the quoted word should be revised as the mental health disorders were mainly identified | The aim of this study is to “identify” the common mental health disorders. | Page 5, line 89 |
|  | Some abbreviations were used in the manuscript which were not specifically defined e.g WHO, PTSD | Corrected | Page 4, line 86  Page 10, line 188 |
|  | There is a need to ensure that adequate information is provided in "Materials and Methods" section e.g information about the contents (sections) in the data collection tool, how the tools was deployed, validated and the parameters used for the sample size calculation. The multi-stage sampling method should be clearly explained | Effected. However, providing most of the requested details would mean repeating what has already being published as details of the methodology were published in a previous paper which was referenced (https://doi.org/10.1177/11786329231166). | Pages 5 line 96 |
|  | Was ethical approval obtained in Ghana? If no, why? | No. The study was an online survey. Ethical approval was sought and obtained from the ethics committee of the institution of the principal investigator and information obtained online from physicians in Nigeria and Ghana. |  |
|  | A non-response rate of over 20% was recorded. What was the reason for this? Can it be included in the limitation? | Included in the limitation | Page 12, line 238 |
|  | In the results section, a chart showing the common adolescent mental health disorder (by country or location of health facility) should be considered (e.g a clustered column bar chart) | Thank you. However, the authors did not consider it necessary as the study was not a comparative study of Nigeria and Ghana rather it was meant to bring to fore the magnitude of adolescent mental health disorders seen by family physicians in both countries and the need for system strengthening to address them. |  |
|  | What further research suggestion on this subject do the authors think should be prioritized? This could be included in the conclusion section. | Done | Page 12, line 229 |
