# Supplementary material for: Common adolescent mental health disorders seen in Family Medicine Clinics in Ghana and Nigeria
Source: PLoS One. 2023 Nov 16;18(11):e0285911. doi: 10.1371/journal.pone.0285911 (PMC10653403; doi:10.1371/journal.pone.0285911)
Supplement: Attachment — Submitted filename: Response to reviewer1.docx [file pone.0285911.s006.docx]

**Response to reviewers**

| Reviewer Number | Original comments of the reviewer | Reply by the author(s) | Changes done on page number and line number |
| --- | --- | --- | --- |
| Reviewer 3 | 1. The method used to determine the 16% burden in this study needs further clarification. It would be beneficial if you could provide a more detailed explanation of how this percentage was calculated. | Clarification provided | Page 7,  Line 138 |
|  | 2. A significant disparity exists between the calculated sample size requirement and the actual distribution of participants between Ghana and Nigeria. Ghana appears to be overrepresented, while Nigeria is notably underrepresented. Moreover, the study's limitation, which attributes this deviation to being an online survey, is a critical concern that could potentially compromise the integrity of the study's conclusions. I strongly suggest further elaboration on this limitation to offer a more comprehensive assessment of its impact on the drawn conclusions. | Reason elaborated more in the limitation | Page 11,  Line 221;  Page 12,  Line 223 |
|  | 3. Epilepsy, also known as a seizure disorder, is classified as a neurological disease rather than a mental health illness. While it's acknowledged that epilepsy can predispose individuals to mental health issues, it remains essential to adhere to accurate classification. This raises questions about the criteria used by physicians to diagnose mental health illnesses within the dataset, particularly since it's listed as the third condition with a frequency of 121 (51.93%). Clarification of the diagnostic criteria for mental health illnesses in this context would be valuable. | Thank you. Epilepsy removed as it is a neurological disorder as highlighted by the reviewer.  It was initially added based on findings of a study on mental and neurological disorders in Ghana. | Page 8,  Line 145, 147  (Table 3);  Page 9,  Line 174;  Page 10,  Line 183 |
| Reviewer 4 | - The statement of the problem needs to be included to the background of the abstract. For instance, what problems could be associated with patients not presenting themselves to mental health physicians? | Included in the abstract | Page 2,  Line 29 |
|  | The justification for the study should also be highlighted. | Justification highlighted. | Page 4,  Line 83 |
|  | Greater clarity is required in the methodology section: - More detail on how the multistage sampling was conducted should be provided | Details of the multistage sampling provided | Page 5,  Line 104 |
|  | -How were the clinic records reviewed to give information about the mental health conditions? This information must be provided? | The records were reviewed by the physicians to confirm the no of mental health patients they have attended to. | Page 6,  Line 113 |
|  | -Even though the authors have indicated that this is part of a larger study, there is still a need for this paper to be able to stand alone and as such there must be at least some information about how the mental disorders examined in this study were assessed e.g. the instruments used to assess each of them and cutoff values. Are these instruments that can be used by any physician or is specialist training required? | Done | Page 5,  Line 104;  Page 6,  Line 113 |
|  | Results -Provide response rate for each country, not only the overall response rate. This would be derived from the proportionate allocation to Ghana and Nigeria respectively. | Country response rate included | Page 6,  Line 126 |
